# Mechanistic insights into the host-microbe interaction and pathogen exclusion mediated by the Mucus-binding protein of *Lactobacillus plantarum*

**DOI:** 10.1038/s41598-018-32417-y

**Published:** 2018-09-21

**Authors:** Kumar Siddharth Singh, Sudarshan Kumar, Ashok Kumar Mohanty, Sunita Grover, Jai Kumar Kaushik

**Affiliations:** 1 Animal Biotechnology Centre, ICAR-National Dairy Research Institute, Karnal, 132001 Haryana India; 2 Dairy Microbiology Division, ICAR-National Dairy Research Institute, Karnal, 132001 Haryana India; 3 BTIS Sub-DIC, Animal Biotechnology Centre, ICAR-National Dairy Research Institute, Karnal, 132001 Haryana India

## Abstract

Surface adhesins of pathogens and probiotics strains are implicated in mediating the binding of microbes to host. Mucus-binding protein (Mub) is unique to gut inhabiting lactic acid bacteria; however, the precise role of Mub proteins or its structural domains in host-microbial interaction is not well understood. Last two domains (Mubs5s6) of the six mucus-binding domains arranged in tandem at the C-terminus of the Lp_1643 protein of *Lactobacillus plantarum* was expressed in *E*. *coli*. Mubs5s6 showed binding with the rat intestinal mucus, pig gastric mucins and human intestinal tissues. Preincubation of Mubs5s6 with the Caco-2 and HT-29 cell lines inhibited the binding of pathogenic enterotoxigenic *E*. *coli* cells to the enterocytes by 68% and 81%, respectively. Pull-down assay suggested Mubs5s6 binding to the host mucosa components like cytokeratins, Hsp90 and Laminin. Mubs5s6 was predicted to possess calcium and glucose binding sites. Binding of Mubs5s6 with these ligands was also experimentally observed. These ligands are known to be associated with pathogenesis suggesting Mub might negotiate pathogens in multiple ways. To study the feasibility of Mubs5s6 delivery in the gut, it was encapsulated in chitosan-sodium tripolyphosphate microspheres with an efficiency of 65% and release up to 85% in near neutral pH zone over a period of 20 hours. Our results show that Mub plays an important role in the host-microbial cross-talk and possesses the potential for pathogen exclusion to a greater extent than mediated by *L*. *plantarum* cells. The functional and technological characteristics of Mubs5s6 make it suitable for breaking the host-pathogen interaction.

## Introduction

Mucus layer of mammalian gut protects against pathogens by shedding off the bound bacteria by peristalsis from the gut^1,2^. Lactic acid bacteria (LAB) are normal inhabitants of mammalian gut and some have been identified as probiotics^3^. Probiotics are live microorganisms which when administered in adequate amounts confer a health benefit to the host^4^. Probiotics have been associated with gut health via modulating the host immune system and the inhibition of pathogens by secreting antimicrobial factors in the gut^3^. Enterotoxigenic *Escherichia coli* (ETEC) is an important gastrointestinal pathogen responsible for bacterial diarrhea throughout the world. ETEC treatment involves broad range antibiotics as the mainline treatment^5^. ETEC and many other gut pathogens employ strategies involving surface adhesins and secretion of toxins to overwhelm host immune system^6,7^. Antimicrobial resistance in bacteria including gastrointestinal pathogens^8^ is ever-increasing and availability of fewer novel antibiotics has worsened the situation. Antimicrobial resistance has prompted intense research to find non-antibiotic based strategies to counter the pathogens. In this context, alternative treatment like microbial interference therapy (MIT) based on adhesion property of probiotics has shown good promise^9^.

Bacterial adhesion holds center-stage in host-microbe interactions and has been proposed to be mediated by surface adhesion proteins like the mucus-binding protein (Mub)^10^, fibronectin binding protein^11^, S-layer protein^12^, collagen binding protein^13,14^ and others. Mucus is a complex viscous mixture of carbohydrates and proteins that provides protection against pathogens by preventing their colonization in the host gut^15^. Almost 70% of total proteins in mucus are represented by various class of mucins, which serve as decoy receptors for microbes. Bacterial adhesins interact with various surface receptors or cytoskeleton proteins of epithelial cell in the gastrointestinal tract (GIT) of the host to mediate bacterial binding. Probiotics and pathogens compete for the common cell receptors for binding with the gut lining in the host’s GIT. Therefore, understanding of the mechanism of host-microbial interaction would pave a way to disrupt the host-pathogen interaction and designing of novel molecules for the pathogen exclusion from the host.

Recently, it has been shown that purified surface adhesion proteins of probiotics can exclude pathogens^16^. The mechanism of bacterial adhesion to host through the surface adhesion proteins is, however, not well understood. In case of Mub this has been primarily because of the large protein size and the structural complexity, ambiguity in the protein domain architecture^1,17,18^ and low level of constitutive expression.

Mub proteins although represented in diverse species^18^ is a peculiar surface adhesion protein restricted to only gut inhabiting species. These proteins contain repetitive Mub domains which are presumed to bind the mucin proteins present in the host mucosa. Mub protein (Lp_1643) from *L*. *plantarum* has interesting architecture in having six tandem Mub domains interspersed by spacer regions^17^. In our earlier study, we cloned, expressed and purified last two domains including spacers (referred to as Mubs5s6) with the maltose binding protein (MBP) tag of this 2200 amino acid residues long protein from an indigenous probiotic *L*. *plantarum* Lp9^19,20^. In this study we report the adhesion of Mubs5s6 protein with different substrata and analyzed the factors which might be involved in its adhesion. To further explore the potential of recombinant Mubs5s6 protein for oral delivery as an antibacterial agent, its stability profile under simulated gastrointestinal conditions was studied and the protective encapsulation was successfully achieved.

## Results

### Purification and muco-adhesive properties of Mubs5s6 protein

**Figure 1:**
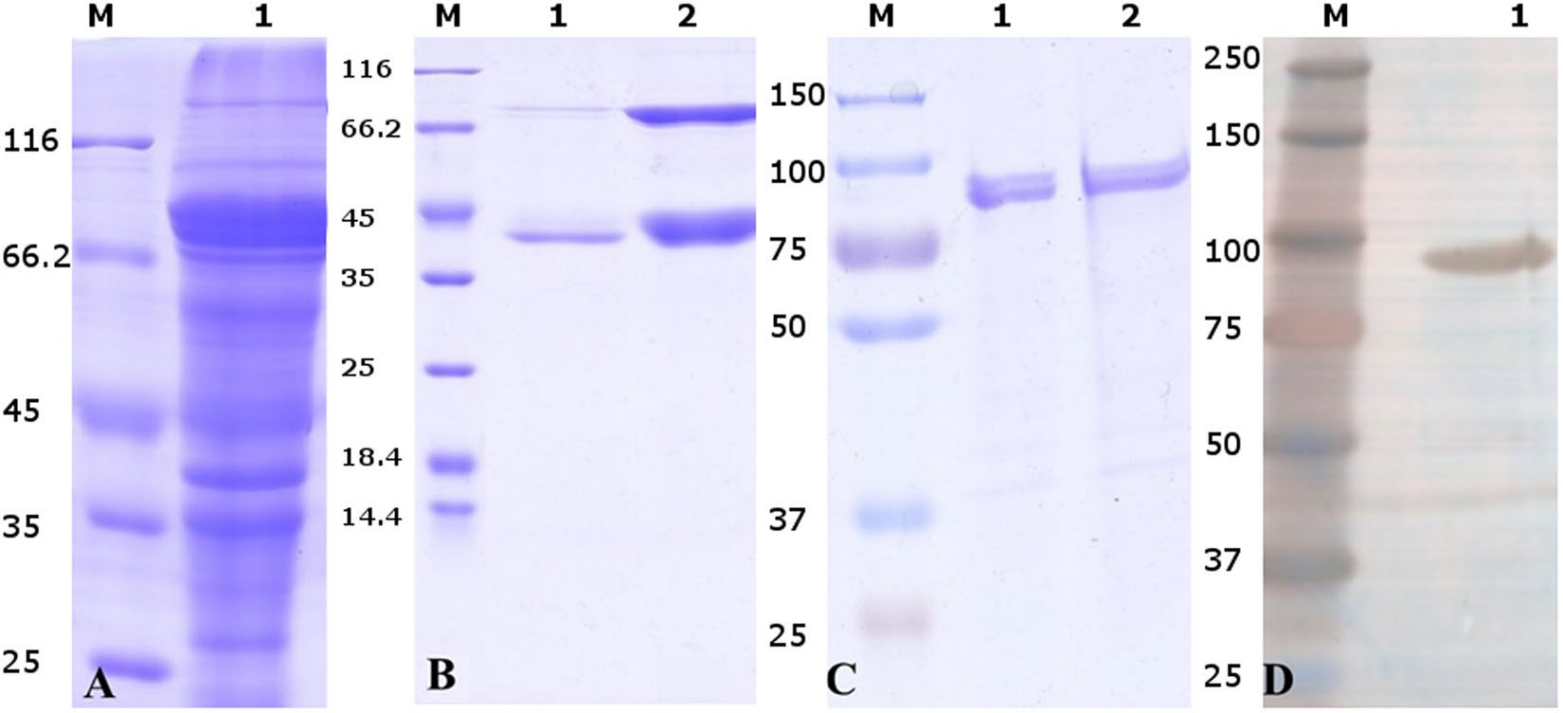
Purification and western blotting of MBP-Mubs5s6 protein. Elution profile of Mubs5s6 purification by (**A**) anion-exchange chromatography: Lane 1 represents the partial purification of MBP-Mubs5s6 protein; (**B**) hydrophobic interaction chromatography, separation of MBP protein from MBP-Mubs5s6 fusion protein preparations: Lane 1 at 50 mM, Lane 2 at 0 mM concentration of (NH_4_)_2_SO_4_; (**C**) Gel Filtration chromatography: Lanes 1 and 2 represent purified MBP-Mubs5s6 protein of size of about 82 kDa; (**D**) Western blot of the purified protein: Lane 1 indicates Mubs5s6 protein. Lane M indicates marker lane.

The clarified cell lysate supernatant containing the soluble 82 kDa MBP-Mubs5s6 fusion protein when used as sample in anion exchange chromatography led to its partial (~50%) purification (Fig. 1A). The partially purified protein was further purified into distinct proteins of sizes 82 kDa (MBP-Mubs5s6) and 42 kDa (MBP tag) eluted at around 50 mM (NH_4_)_2_SO_4_ from the phenyl-sepharose column (Fig. 1B). Gel filtration purification of the MBP-Mubs5s6 protein resulted in a single peak suggesting the homogeneous preparation of the protein (Fig. 1C). The MBP-Mubs5s6 protein was confirmed with western blotting (Fig. 1D).

Earlier purification of Mubs5s6 protein by amylose resin affinity chromatography resulted in low yield^19^ and significant batch to batch variations. In the current protocol, the final yield of Mubs5s6 protein was still small, but the semi-automation of the purification resulted in the reproducibility of the process.

**Figure 2:**
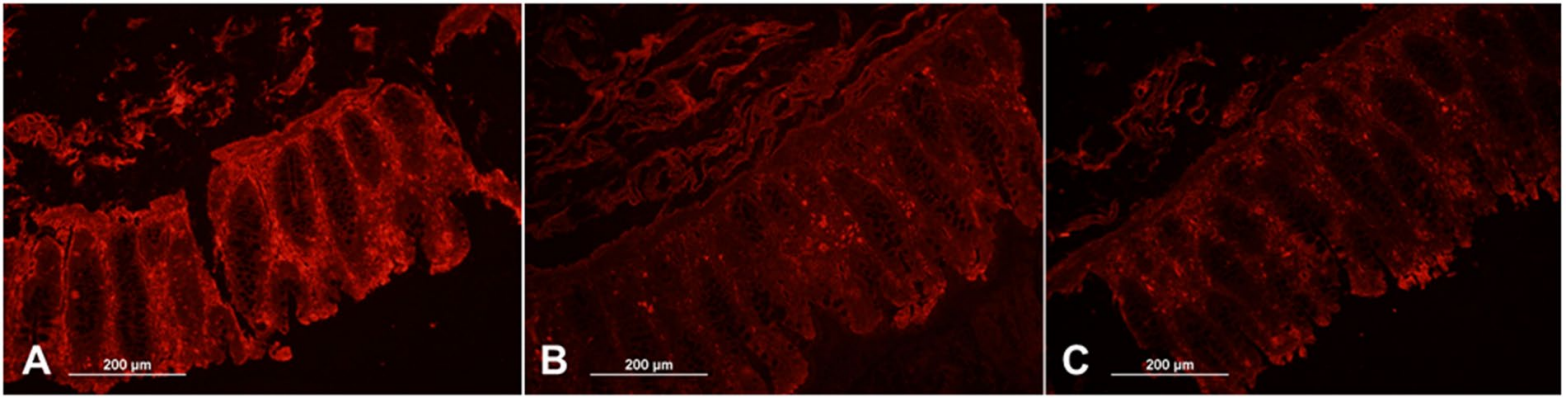
Binding of Mubs5s6 protein to the human intestinal tissue sections. Tissue binding signal in the form of immunofluorescence was detected in the presence of buffers at pH (**A**) 7.4, (**B**) 5.0 and (**C**) 3.5.

The strong adherence of MBP-Mubs5s6 protein to the human intestinal tissue sections^19^ was visibly decreased at low pH (≤5) solution conditions, while no significant effect was observed at pH 7.4 (Fig. 2). This might be ascribed to the denaturation of Mubs5s6 protein at low pH as observed during its incubation at low pH (Supplementary Table S2). Mubs5s6 protein showed binding to the mice intestinal mucus, immobilized PGM and human intestinal cell lines HT-29 and Caco-2. The binding capacity of Mubs5s6 (7 µg) decreased by 35.36 ± 1.07 (% decrease ± SEM), 11.03 ± 1.24, 33.89 ± 1.96 and 64.63 ± 1.01 after exposure to simulated gastric fluid (SGF), simulated intestinal fluid (SIF), bile salts (BS) and Tween-20, respectively. All these observations showed %SEM below 2.1 and coefficient of variation for different groups within the acceptable limits of 15% (Supplementary Table S1).

### Binding of Mubs5s6 with Mucin-III and other cellular proteins and small ligands

**Figure 3:**
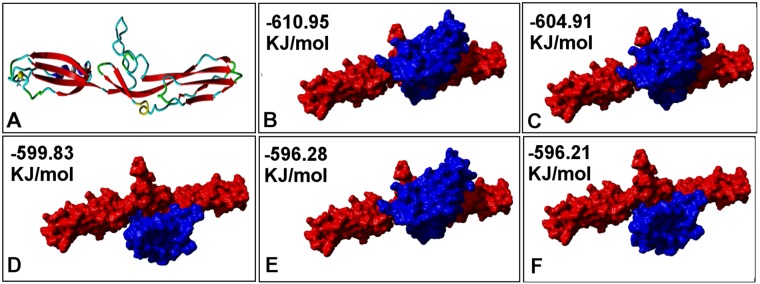
Structural models of Mubs5s6 protein and complexes with Mucin-III. Homology model of (**A**) Mubs5s6 protein; (**B–F**) predicted complexes between Mubs5s6 (shown in red color) and Mucin-III (blue color). All top docking poses indicate that Mucin-III is binding near the middle region of Mubs5s6 protein. The predicted interaction energies are also shown in each panel.

Out of five top hits, 3i57B was chosen as the template for homology model prediction of the largest stretch (208 amino acids) of Mubs5s6 protein (366 amino acids). The homology model of Mubs5s6 suggested it to be constituted of mostly beta-sheets (Fig. 3A), which is consistent with the fact that mucus binding proteins have immunoglobulin like fold^21^. This model was used for docking with the predicted model of Mucin-III molecule. HEX program generated top five complexes with highest predicted interaction energies are shown in Fig. 3B–F. Similar docking complexes were also obtained among the top solutions by HADDOCK program^22^ suggesting high probability of these solutions.

By using the pull-down assay followed by LC-MS/MS based analysis of HT-29 and Caco-2 cell surface proteins having affinity for Mubs5s6 we could identify cytokeratins (1, 5, 6, 9, 10) in >60 kDa size fraction, cytokeratins (1, 6, 9, 10) in 60–45 kDa size fraction and cytokeratins (1, 4, 5, 6, 8, 9, 10) in the 45–25 kDa size fraction. We also confirmed the presence of low amounts of laminin and Hsp90 in the fraction size at >60 kDa by using western blot. Predictions by the COACH server^23^ suggested that Mubs5s6 possesses potential binding sites for calcium (Asp59, Val60, Aap61, Phe63 and Phe64) and glucose (Asn42, Gly43, Asn224, Thr227, Asn228 and Gln229). We could also confirm the binding of calcium and glucose by Atomic Absorption Spectroscopy and biochemical assay, respectively (as described in Supplementary information).

### Pathogen exclusion by Mubs5s6 protein

Pre-incubation of Mubs5s6 protein (400 µg/ml) with cultured intestinal cell lines inhibited the adhesion of ETEC to HT-29 and Caco-2 cell lines by 81 ± 0.8% and 68 ± 0.7, respectively, in comparison to the control. The pathogen adhesion decreased with increasing Mubs5s6 concentration. At the lowest concentration of 25 µg/ml used in this study, Mubs5s6 inhibited ETEC binding by as high as 33 ± 0.7% (Table 1). We did not observe any significant change (~2%) in the viability of the HT-29 and Caco-2 cell lines upon incubation with the recombinant Mubs5s6 protein as high as 500 µg/ml. These results showed that the Mubs5s6 protein used in the study was completely free from endotoxin, which might otherwise be carried over during purification from *E*. *coli* lysate. We also used *L*. *plantarum* Lp9, which was used to clone Mubs5s6, as a positive control to inhibit ETEC binding with enterocytes. Interestingly, at *L*. *plantarum* Lp9 cell count of 10^6^/ml the ETEC binding to HT-29 and Caco-2 cells was inhibited by 50% and 37%, respectively (Table 1). These results showed that the isolated proteins could be more effective than the microbial organisms.

**Table 1:** Decrease in the adhesion of enterotoxigenic *E*. *coli* (ETEC) to HT-29 cells and Caco-2 cells in the presence of Mubs5s6 protein or *L*. *plantarum* Lp9, n = 6.

Sl. No	Cell line: Mubs5s6 or *L*. *plantarum* Lp9: ETEC	%Decrease ± SEM
1	HT-29: No protein or Lp9: ETEC (control)	0
2	HT-29: Mubs5s6 (25 µg/ml): ETEC	33.26 ± 0.7
3	HT-29: Mubs5s6 (125 µg/ml): ETEC	53.79 ± 0.9
4	HT-29: Mubs5s6 (400 µg/ml): ETEC	81.59 ± 0.8
5	HT-29: Lp9: ETEC	49.86 ± 1.0
6	Caco-2: No protein or Lp9: ETEC (control)	0
7	Caco-2: Mubs5s6 (25 µg/ml): ETEC	24.18 ± 0.6
8	Caco-2: Mubs5s6 (125 µg/ml): ETEC	39.60 ± 0.8
9	Caco-2: Mubs5s6 (400 µg/ml): ETEC	68.00 ± 0.7
10	Caco-2: Lp9: ETEC	36.56 ± 0.9

The probiotic *L*. *plantarum* Lp9 and pathogen ETEC cells were used at 10^6^ CFU/ml.

### Stability and encapsulation of recombinant Mubs5s6 protein

The thermal denaturation profile of Mubs5s6 protein (0.5 mg/ml) showed that the Mubs5s6 protein was stable at 37 °C in the alkaline pH region; however the protein aggregated at pH ≤ 5.0 necessitating the measurement of the thermal denaturation at pH 7.4 to pH 10 (Supplementary Table S2). The protein was observed to be stable at physiological condition. The pathogen exclusion experiments were also carried out at pH 7.4 where the protein should be in native state. On the other hand, the MBP-Mubs5s6 protein suffered a loss in its adhesion to PGM when exposed to SGF (by 35.36 ± 1.07%), SIF (11 ± 1.24%), BS (33.89 ± 1.96%) in comparison to the adhesion of intact MBP-Mubs5s6 protein.

To protect the protein, the Mubs5s6 protein was encapsulated in pH-responsive chitosan and sodium tri-polyphosphate (TPP) microspheres. The microspheres prepared with 1.75% chitosan, 2% TPP and 500 µg of Mubs5s6 protein showed 65% encapsulation efficiency with desired spherical shape and protein release behavior. The microspheres released up to 85% of protein at alkaline pH over a period of 20 hours in a controlled manner (Supplementary Figure S5). The released Mubs5s6 protein showed no significant difference in its adhesion to the intestinal tissues and the efficiency to exclude pathogens from the HT-29 and Caco-2 cell lines (Supplementary Table S3).

## Discussion

Recombinant Mubs5s6 possessing only last two domains out of total six Mub domains at the C-terminal of the Lp_1643 protein of *L*. *plantarum* Lp9 has been shown to adhere with human intestinal tissue sections^19^. Mubs5s6 did not bind to ‘mucus globules’ of intestinal tissue sections. They could rather bind to the extracellular matrix (in basal layer) and microvilli apical surface which contains surface-bound mucins, fibronectin, collagen and other surface proteins^24^. This shows that Mubs5s6 either fails to access the secretory globules or is inherently unable to adhere with these specific types of intestinal secretory mucins. This is an interesting behavior, since it binds to PGM and mice mucus. There are few reports of selective adhesion where a bacterial surface protein binds to only a specific type of mammalian surface protein^25^.

### Hydrophobic interactions dominate the binding of Mubs5s6 with gut mucosa components

In some cases, electrostatics is known to play an important role in modulating the behavior of surface adhesion proteins primarily due to disturbed ionic interactions^26^. Spa B, a surface protein of *L*. *rhamnosus* GG having pI = 8, has been shown to adhere to negatively charged mucus *via* electrostatic interactions^27^. Mubs5s6, an acidic protein (pI of 4.9), was able to bind to acidic mucus and PGM (pI < 5) at high pH, where they should otherwise repel. This suggested that electrostatic forces might not be responsible for binding of Mubs5s6 to mucus, PGM and possibly intestinal tissue. The adhesion of Mubs5s6 to the tissue sections got considerably reduced when pH of the binding buffer was decreased below 5.0. We also observed Mubs5s6 forming insoluble aggregates below pH 5.0 suggesting that the decrease in the binding could be due to Mubs5s6 aggregation. The mucus components are known to interact with the charged (sialic acids), hydrophilic (carbohydrates such as N-acetylgalactosamine, fucose) and the hydrophobic surfaces (non-glycosylated Cys-rich hydrophobic regions of protein backbone)^28,29^. Addition of the Tween-20 decreased the binding of Mubs5s6 to the PGM, which suggests that the interaction between Mubs5s6 and PGM might be mediated by the hydrophobic interactions.

Pathogenic *E*. *coli* surface adhesins utilize hydrophobic interactions, cation-bridging and receptor-ligand interactions to mediate adhesion with the host gut^30,31^. Higher surface hydrophobicity of the probiotic strains has been associated with stronger adhesion to host^32^ and better competitive inhibition of pathogens^33^. We also observed the dominance of hydrophobic interactions in the binding of Mubs5s6 to PGM. Therefore, it seems that hydrophobic interaction although non-specific in nature is important in host-microbe interaction through specific protein-protein interactions.

### Mubs5s6 possesses affinity for surface and cytoskeleton proteins of the gut epithelial cells

The Mubs5s6 protein showed strong binding with two intestinal cell lines Caco-2 and HT-29 (Supplementary Figure S3 and S4). Caco-2 mainly expresses surface bound mucins such as MUC3^34^. It seems that the Mubs5s6 protein binds to both the surface bound mucins and other surface and cytoskeleton proteins in Caco-2 cells, since immunofluorescence was observed throughout the cell surface. The pull-down assay also suggested the binding of the Mubs5s6 protein with several cytokeratins. Recently, a Mub protein has been shown to bind freshly trypsinized free-floating Caco-2 cells^35^, suggesting that the Mub protein has the affinity for the cytoskeletal proteins like cytokeratins as well. The HT-29 cell line mainly expresses secretory mucins such as MUC2 and MUC5AC on its surface^36^. Some adhesins like GroEL of *L*. *johnsonii* NCC533^37^, LspA of *L*. *salivarius* UCC118^38^, and Mub of *L*. *reuteri*^39^ have been demonstrated to bind the HT-29 cell monolayer. Probiotic organisms show differential binding behavior towards cell matrix components like mucins (PGM)^40^, basement membrane^41^ and collagen^14^. These results support our results suggesting that the probiotics-host cross talk is mediated by the probiotic surface proteins and the extracellular matrix proteins as well as the cytoskeletal proteins of the host.

MS/MS results also showed binding of other proteins like Hsp90 (2 peptides) and Laminin (1 peptide) with Mubs5s6, although with lower reliability. However, western blotting confirmed the presence of these proteins in the eluted fraction (Supplementary Figure S6). The Hsp family proteins (65, 70 and 90 kDa size) are known to serve as receptors for the bacterial adhesion^42,43^ and some (gp96 or Hsp90B1) of these are vital for the bacterial stimuli^44^. Laminin has also been shown to act as a receptor for many bacteria including probiotics^45^. Mucins were not detected in the bound fraction because loosely attached secreted mucins were removed in the pre-washing steps during the isolation of the surface proteins from the HT-29 and Caco-2 cells.

### Mubs5s6 is more effective than the source probiotic in preventing pathogen adhesion

Since adhesion holds prime importance in pathogenicity^46^, interactions of Mubs5s6 protein with mucus or cell surfaces were expected to prevent colonization of intestinal pathogens. Adhesion of ETEC decreased with the increasing concentration of Mubs5s6 protein. Previous studies have also shown high pathogen exclusion (48–95%) by using bacterial surface adhesins^35,47^. It seems logical to think that whole probiotic cells might be more efficient in pathogen exclusion due to their large size and the presence of diverse kinds of surface adhesins. On the contrary, we observed that preincubation of *L*. *plantarum* Lp9 could decrease pathogen adhesion to a much lower extent when compared to purified Mubs5s6 protein (Table 1). Previous reports have shown that the probiotic organisms could inhibit ETEC binding to the cultured intestinal cells by as high as 60%^33,48^. In our case, higher pathogen exclusion by Mubs5s6 protein might be attributed to its freely diffusible smaller size and hence better accessibility of cell receptors resulting in the blanketing of the cells and tissues (Fig. 2) to preclude the binding of pathogens.

### Mubs5s6 is a multifunctional protein with glucose and calcium binding properties

*In silico* prediction followed by experimental validation confirmed the specific binding of calcium and glucose to Mubs5s6 protein. Calcium is an important intracellular messenger; however, its extracellular presence has been linked with the adhesion of pathogens including *E*. *coli* to host cells^49,50^. Interestingly, dietary calcium has been shown to specifically control ETEC mediated diarrhea in humans^51^. Glucose on the surface of bacteria has been directly associated with the adherence of ETEC pathogen^52^ and also in manifesting pathogenicity of *Y*. *pestis*^53^. Few bacterial surface proteins have been shown to possess moonlighting attributes and putative roles other than the functional role they have been classified^26,54^. It is possible that calcium and glucose might be mediating the adhesion of bacterial species including the pathogens and probiotic bacteria to host cells. In case of *L*. *plantarum*, calcium and glucose molecules might be held by Mub protein on the surface and thus modulating the binding with the host cells. However, further work is required to confirm it. The binding of several proteins and small molecules with Mubs5s6 suggest it to be a multifunctional protein perhaps with moonlighting attributes.

Even though Mubs5s6 has high pathogen exclusion potential, it suffered loss in adhesion to the PGM upon exposure to low pH, SGF and bile salts. On the other hand, the decrease in adhesion was smaller over exposure to SIF. For delivery to GIT, the protein was encapsulated in the biocompatible chitosan-TPP^55,56^ that released Mubs5s6 protein in a controlled manner. Efficient release of Mubs5s6 protein from the microspheres in the neutral-alkaline pH prevalent in the intestine should help in the exclusion of pathogens from the gut.

In summary, truncated Mub protein (Mubs5s6) of the Lp_1643 of *L*. *plantarum* Lp9 showed binding to different gut mucosa components, human enteric tissues and their surface components like cytokeratins, laminin and Hsp90. Probiotic Mubs5s6 also showed affinity for the calcium and glucose molecules, which are involved in the pathogenesis. Mubs5s6 reduced the pathogenic ETEC binding to the human enterocytes, which is the first step in the pathogenesis. Hydrophobic interactions dominated the binding of Mubs5s6 to porcine gastric mucin. These results shed light on the poorly understood mechanism of adhesion during host-microbe interactions. High pathogen exclusion activity and efficient encapsulation makes Mubs5s6 protein suitable for developing it as an effective prophylactic or a therapeutic agent against gut pathogens.

## Methods

### Expression and purification of Mubs5s6 protein

The Mubs5s6 gene cloned and expressed in *E*. *coli* Lemo host^19^ as fusion protein with maltose binding protein (MBP) tag was purified by non-affinity based chromatography to improve the yield. The crude lysate of induced *E*. *coli* culture was equilibrated with binding buffer (50 mM Tris-HCl, pH = 7.5) by using HiPrep 26/10 desalting column. The lysate was purified by using the Q-Sepharose HiLoad 16/10 anion exchanger column connected with AKTAexplorer. The linear gradient was applied with elution buffer containing 1 M NaCl, 50 mM Tris-HCl, at pH 7.5. The Mub containing fractions were further purified by using HiLoad 16/10 Phenyl-Sepharose hydrophobic interaction chromatography and applying a linear gradient of binding buffer (1 M (NH_4_)_2_SO_4_, 100 mM Na_2_HPO_4_, pH 7.5) and the same buffer without (NH_4_)_2_SO_4_ as elution buffer. Finally Superdex 75 10/300GL gel filtration column was used for obtaining highly purified Mubs5s6 protein. The GE Healthcare chromatography hardware and software were used in all the steps of purification. The identity of purified protein was confirmed by western blot as described previously^19^. The purified 82 kDa MBP-Mubs5s6 was stored as 100 µl aliquots in storage buffer (50 mM Tris, 0.1 M NaCl, 20% Glycerol, 0.5 mM PMSF, pH 7.5) at −20 °C for the immediate use or flash-frozen in liquid nitrogen and kept at −80 °C for long term storage.

### Mub adhesion with host mucosa components

The adhesion of MBP-Mubs5s6 protein with human intestinal tissue sections was studied at varying pH values (2.5, 5 and 7.4) as previously described^19^. Small intestine mucus from a sacrificed mice and porcine gastric mucin (PGM) were separately and mildly heat-fixed on glass slides and incubated with 100 µl of 250 µg/ml MBP-Mubs5s6 protein for 1 hour. The slides were washed off with phosphate buffer saline (PBS) and then incubated with anti-MBP rabbit primary antibody (0.1 µg/ml) overnight at 5 °C followed by three times washing with PBS. It was then incubated with phycoerythrin tagged anti-rabbit goat secondary antibody (0.2 µg/ml) for 2 hours at room temperature. The slides were again washed thrice with PBS, air-dried and visualized under the fluorescence microscope with green filter (BX-51, Olympus, Japan). Purified MBP protein was used as a control in the binding assays. In all the adhesion experiments, substrata were blocked with 2% BSA in PBS for 4 h, washed with PBS and then incubated with MBP-Mubs5s6 protein. Also, 1% BSA was used in the primary and secondary antibody solutions.

### Adhesion with human intestinal cell lines

Caco-2 and HT-29 cell lines procured from NCCS, Pune, India were seeded (1 × 10^4^ cells/ml) in DMEM (Dulbecco’s Modified Eagle’s Medium, supplemented with 4500 mg/L Glucose, 26 mM NaHCO_3_, 2.5 mM L-Glutamine, 20% heat-inactivated Fetal Bovine Serum (FBS), 1% non-essential amino acids, 100 µg/ml Penicillin and 100 U of Streptomycin) over glass coverslips placed in 12-well cell culture plate and incubated at 37 °C, 5% CO_2_ in a CO_2_ incubator. The culture plate was monitored daily and replaced with fresh media (DMEM with 10% FBS) every two days. The spent media was removed and the culture plate was washed thrice with minimal DMEM (No FBS, No Antibiotic and No Glutamine). The 80–90% cell confluence was observed after 21 and 15 days in Caco-2 and HT-29 cells, respectively, and their monolayer was used after another 5 and 10 days, respectively, to allow the expression of surface proteins by these monolayers. The spent media was removed and the culture plate was washed thrice with minimal DMEM (No FBS, No Antibiotic and No Glutamine). Control wells seeded with respective cell lines were used for measuring cell viability with the standard Trypan blue exclusion assay^57^.

The wells in quadruplicates were incubated for 60 min with 300 µl of 250 µg/ml endotoxin-free MBP-Mubs5s6 protein, prepared by using the endotoxin removal kit (Thermo Fisher, USA). The plate wells were washed thrice with minimal DMEM and fixed with 100% methanol for 15 minutes. The bound protein was visualized using immunofluorescence at 20X and 40X under an inverted fluorescent microscope. MBP protein which served as a detection tag in MBP-Mubs5s6 fusion protein was purified and used separately as a negative controls for comparison.

### Quantitative estimation of adhesion with porcine gastric mucin

The purified MBP-Mubs5s6 fusion protein was assessed for its adhesion ability with porcine gastric mucin (PGM) which contains Mucin-III (Sigma, USA). 100 µl of 200 µg/ml solution of PGM in 100 mM sodium bicarbonate/carbonate buffer (3.03 g/L Na_2_CO_3_, 6.0 g/L NaHCO_3_, pH 9.6) was left overnight (12–16 hours) at 5 °C for passive immobilization of mucin proteins on a 96 well polystyrene ELISA plate. The plate was then washed thrice with PBS, blocked with 1% BSA for 4 hours at 25 °C, washed thrice with PBS and incubated with 100 µl of varying concentrations (5–250 µg/ml) of MBP-Mubs5s6 protein for 1 hour on an orbital shaker. The wells were washed thrice with PBS and blocked with 2% BSA dissolved in PBS for 5 hours at 25 °C. Thereafter, the plate was washed thrice with PBS and incubated with anti-MBP rabbit primary antibody (1:20000 dilution/0.5 × 10^−4^ µg) for 4 hours at 25 °C.

The plate was washed off thrice with PBS and then incubated with HRP conjugated anti-rabbit goat secondary antibody (1:10000 dilution/1 × 10^−4^ µg) for 2 hours at 25 °C followed by washing three times with PBS and incubation with 50 µl of 3,3′,5,5′-Tetramethylbenzidine (TMB) substrate for 1–5 min. When blue color appeared, the reaction was stopped with 20 µl of 2 M HCl resulting into appearance of yellow color. The intensity of the coloration was measured at 450 nm with Nano Quant M200 Pro ELISA Reader (Tecan, Finland). Separated MBP and PBS were used as negative controls for comparison. The binding of MBP-Mubs5s6 exposed to gastrointestinal conditions (SGF, SIF and BS) to PGM was also measured by same method.

To measure the contribution of hydrophobic interactions in the binding of Mubs5s6 with Mucin-III, we mixed 2% Tween-20 with MBP-Mubs5s6 and allowed binding at room temperature. Both MBP-Mubs5s6 and PGM were found to be stable in presence of 2% Tween-20, as observed prior to this experiment. All these experiments were carried out twice in triplicate each time and data were expressed as the percentage difference (±SEM) between the absorbance signal before and after treatment with Tween-20, calculated as follows:

$${\rm{Change}}\,{\rm{in}}\,{\rm{binding}}( \% )=[{\rm{Abs}}({\rm{Initial}}-{\rm{Final}})/{\rm{Initial}}]\times 100$$

The percent change in binding because of the treatment with Tween-20 was taken as a measure of contribution of hydrophobic interactions in the binding of proteins.

### Bioinformatics analysis and probable binding partners for Mubs5s6 protein

The tertiary (3-D) structure of Mubs5s6 was predicted by homology modelling based on experimentally available template structures for Mub proteins of Lactobacillus origin. Yasara-Structure suite^58^ and Swiss Model webserver^59^ were used to align the Mubs5s6 primary amino acid sequence with sequences of 3-D structures available in RCSB database and the top hit (3i57B, 1.8 Å resolution) was chosen as template. A homology model was generated and the final model was energy minimized with FoldX program. The model was refined with Modrefiner^60^ and the stereochemical quality of the model was checked by Verify 3D^61^.

The generated Mubs5s6 model and Mucin-III structure^62^ (2OYP_A) were used for *in silico* rigid body molecular docking with Hex software. Top five hits with lowest predicted interaction energies were chosen to analyze as best docking complexes. The model of Mubs5s6 was also used for searching its potential binding partners using COACH tool of I-Tasser suite^23^.

### Pull down assay for the identification of binding partners of Mub protein

Surface or membrane proteins of the HT-29 and Caco-2 intestinal cell lines were isolated by using the protocol described by Howell and coworkers (1992)^63^ with slight modifications. Cells were resuspended in 20 mM Tris-HCl binding buffer at pH 7.4. To remove the 42 kDa MBP tag from the ~82 kDa MBP-Mubs5s6 fusion protein, the purified protein was incubated with Factor Xa (Cat. No. P8010S, NEB, USA) in a ratio of 100:1 for 8 hours at 23 °C. The undigested (MBP-Mubs5s6) and the cleaved proteins (MBP-Mubs5s6 and Mubs5s6) were separated on a gel filtration column. The 40 kDa Mubs5s6 protein was separated from the MBP tag by anion exchange chromatography. Mubs5s6 protein (0.8 mg/ml) was covalently bonded with 2 ml NHS-activated Sepharose 4B beads (GE Healthcare, USA). The matrix was packed in a column pre-equilibrated with binding buffer and the extracted surface proteins were allowed to bind at a flow rate of 0.2 ml/min. The column was washed thrice with binding buffer to remove loosely bound proteins followed by elution of bound proteins with citrate buffer (pH 5.0) supplemented with 2% Tween-20.

The eluted proteins were concentrated and separated with SDS-PAGE. Based on their sizes on gel, the separated proteins were divided in three fractions: 25–45 kDa, 45–60 kDa and >60 kDa. Protein samples were prepared and analyzed with Maxis-HD qTOF (Bruker Daltonics, Germany) LC-MS/MS as described earlier^64^. The MS/MS spectra were analyzed with the BioTools 2.2 software package and the MASCOT search engine to identify proteins. Western blot was used to confirm the proteins identified with low reliability (Laminin and Hsp90).

### Pathogen exclusion by Mubs5s6 protein

To investigate pathogen exclusion by Mubs5s6 protein, the cultured cell monolayers (Caco-2 and HT-29) were incubated with varying concentrations (25–400 µg) of endotoxin-free and MBP tag free Mubs5s6 protein for one hour. The protein was washed off and the wells were washed thrice with minimal DMEM followed by incubation for one hour with 300 µl of 10^5^–10^7^ CFU/ml of enterotoxigenic *E*. *coli* (ETEC) pathogen (MTCC-IMTECH, India). The wells were washed off thrice with minimal DMEM and trypsinized to recover all cells along with adherent bacteria. This mixture was serially diluted and plated on LB agar supplemented with 0.5% Glucose^65^. The overnight grown colonies were counted and compared with control batches where cells were not incubated with Mubs5s6 protein prior to incubation with ETEC. Experiments were carried out twice with three replicates each time. The toxicity of Mubs5s6 protein on cells viability was measured with Trypan blue exclusion assay^57^ after incubation of the confluent and trypsinized cells with 50–500 µg/ml Mubs5s6 protein. The probiotic *L*. *plantarum* Lp9, the source of Mubs5s6 gene, was also used at 10^6^ CFU/ml (300 µl) for pathogen inhibition for comparison with Mubs5s6 protein.

### Determination of the thermal stability of Mubs5s6 protein

A melting curve of Mubs5s6 was measured by linearly increasing the temperature and measuring the change in absorbance at 280 nm on a spectrophotometer (UV-2600, Shimadzu, Japan) fitted with a peltier temperature controller. The 1^st^ derivative of the data points was used to calculate the midpoint of melting (T_m_). To observe the effect of pH, Mubs5s6 protein was dissolved separately in 20 mM Glycine-HCl, pH 2.0; 20 mM acetate buffer, pH 5.0; and 20 mM Glycine-NaOH, pH 9.5.

### Determination of the stability of Mubs5s6 under the simulated gut conditions

Before exposure to simulated gastrointestinal conditions, the pH of MBP-Mubs5s6 fusion protein buffer was set at pH 2 (similar to stomach), pH 6.8 (similar to intestine) and high pH (10.0) as a preliminary check for denaturation. In a separate experiment, MBP-Mubs5s6 protein (100 µg, 50 µl) was incubated with 50 µl of simulated gastric fluid (35 mM NaCl, 0.25% HCl, 0.32% Pepsin, pH 2) at 37 °C for 45 minutes^40^. The pepsin in the mixture was then inactivated by increasing the pH to ~7.0 using 50 µl of 0.25% NaOH. The pH was raised to avoid denaturation of the immobilized PGM for assessing the residual binding of Mubs5s6 after treatment with SGF by indirect ELISA method. Similarly, the protein was studied for residual adhesion potential after exposure to simulated intestinal fluid (0.68% K_2_HPO_4_, 15.4 mM NaOH, 10% pancreatin, pH 6.8), or bile salts mixture (Sigma, USA) (0.68% K_2_HPO_4_, 15.4 mM NaOH, 0.3% Bile salts mixture) for 3 hours at 37 °C^40^. The results were compared with quantitative adhesion with PGM to assess the decrease in adhesion due to SGF or SIF treatment. Purified MBP was used as control in all these experiments. Prior to these experiments, PGM stability was checked over exposure to SGF, SIF or Bile salts.

### Encapsulation of Mubs5s6 protein and its controlled release

The Mubs5s6 protein was encapsulated in pH-responsive Chitosan and sodium tri-polyphosphate (TPP) microspheres. One mg of Mubs5s6 protein was mixed with 10 ml of 1.5% chitosan (dissolved in 1% acetic acid) and dropped in a 2% aqueous TPP solution while being gently stirred. After 30 minutes, the microspheres were taken out, rinsed with distilled water and air-dried for 24 hours at room temperature. It was then dried in hot-air oven for 3 hours at 37 °C and stored in air-tight plastic pouches. The amount of encapsulated protein was checked by crushing the microspheres in double distilled water and estimating the amount of Mubs5s6 protein using Bradford reagent (Merck, USA), taking BSA (Amresco, USA) as a standard. In another experiment, the microspheres were resuspended in 1 ml HCl (pH 1.2) buffer and kept for 2 hours with shaking (100 rpm) at 37 °C and then washed twice with distilled water. These microspheres were resuspended in 1 ml of phosphate (pH 6.8) buffer and left for 18 hours at 37 °C to estimate the amount of Mubs5s6 released. The released protein was also checked for pathogen exclusion potential on HT-29 and Caco-2 cell lines as explained in previous sections.

### Ethical statement and recombinant DNA regulations

The study was approved by the Institutional Animal Ethics Committee of ICAR-National Dairy Research Institute, Karnal, India and the Committee for the Purpose of Control and Supervision of Experiments on Animals (Sanction No. 1705/GO/ac/13/CPCSEA Dated: 3 July 2013), Ministry of Environment, Forest and Climate Change, Govt. of India. All the experiments involving recombinant techniques were approved by the Institute Biosafety Committee under the guidelines of Review Committee on Genetic Manipulation, Department of Biotechnology, Govt. of India, New Delhi, India.

## Electronic supplementary material

Supplementary information

## Data Availability

All relevant data are within the paper and its Supporting Information files.

## References

[1] Roos S, Jonsson H (2002). A high-molecular-mass cell-surface protein from Lactobacillus reuteri 1063 adheres to mucus components. Microbiology..

[2] Curtis M, Sperandio V (2011). A complex relationship: the interaction among symbiotic microbes, invading pathogens and their mammalian host. Mucosal Immunology..

[3] Pagnini C (2010). Probiotics promote gut health through stimulation of epithelial innate immunity. Proceedings of the National Academy of Sciences USA.

[4] Report of a Joint *FAO/WHO* Working Group on Drafting Guidelines for the Evaluation of Probiotics in Food. London, Ontario, Canada. April 30 and May 1 (2002).

[5] Kotloff KA (2013). Burden and aetiology of diarrhoeal disease in infants and young children in developing countries (the Global Enteric Multicenter Study, GEMS): a prospective, case-control study. The Lancet..

[6] Canto DF (2012). Identification of coli surface antigen 23, a novel adhesin of Enterotoxigenic Escherichia coli. Infection and Immunity..

[7] Li Y (2007). A receptor-binding site as revealed by the crystal structure of CfaE, the colonization factor antigen I fimbrial adhesin of Enterotoxigenic Escherichia coli. Journal of Biological Chemistry..

[8] Tytgat H (2016). Lactobacillus rhamnosus GG outcompetes Enterococcus faecium via Mucus-Binding pili: evidence for a novel and heterospecific probiotic mechanism. Applied and Environmental Microbiology..

[9] Asahara T (2004). Probiotic Bifidobacteria Protect Mice from Lethal Infection with Shiga Toxin-Producing Escherichia coli O157:H7. Infection and Immunity..

[10] Tassell VM, Miller M (2011). Lactobacillus Adhesion to Mucus. Nutrients..

[11] Bisht S (2018). Expression of fibronectin-binding protein of L. acidophilus NCFM and in vitro refolding to adhesion capable native-like protein from inclusion bodies. Protein Expression and purification..

[12] Li P, Yu Q, Ye X, Wang Z, Yang Q (2011). Lactobacillus S-layer protein inhibition of Salmonella-induced reorganization of the cytoskeleton and activation of MAPK signaling pathways in Caco-2 cells. Microbiology..

[13] Yadav AK (2013). Role of surface layer collagen binding protein from indigenous Lactobacillus plantarum 91 in adhesion and its anti-adhesion potential against gut pathogen. Microbiological Research..

[14] Salzillo M (2015). Identification and characterization of enolase as a collagen-binding protein in Lactobacillus plantarum. Journal of Basic Microbiology..

[15] Parker P (2010). Bovine Muc1 inhibits binding of enteric bacteria to Caco-2 cells. Glycoconjugate Journal..

[16] Du L, Liu F, Ju X, Huo G (2010). Adhesion capability of first two domains at N terminus of NP_785232 protein and their interaction with a UV-absorbing component from human mucus. Letters in Applied Microbiology..

[17] Boekhorst J, Wels M, Kleerebezem M, Siezen R (2006). The predicted secretome of Lactobacillus plantarum WCFS1 sheds light on interactions with its environment. Microbiology..

[18] Kleerebezem M (2010). The extracellular biology of the lactobacilli. FEMS Microbiology Reviews..

[19] Singh K (2017). Expression of recombinant truncated domains of mucus-binding (Mub) protein of Lactobacillus plantarum in soluble and biologically active form. Protein Expression and Purification..

[20] Kaushik JK (2009). Functional and probiotic attributes of an indigenous isolate of Lactobacillus plantarum. PLoS ONE..

[21] MacKenzie DA, Tailford LE, Hemmings AM, Juge N (2009). Crystal structure of a mucus-binding protein repeat reveals an unexpected functional immunoglobulin binding activity. Journal of Biological Chemistry..

[22] Zundert VGCP (2016). The HADDOCK2.2 webserver: User-friendly integrative modeling of biomolecular complexes. Journal of Molecular Biology..

[23] Roy A, Yang J, Zhang Y (2012). COFACTOR: an accurate comparative algorithm for structure-based protein function annotation. Nucleic Acids Research..

[24] Hynes RO, Naba A (2012). Overview of the Matrisome - An Inventory of Extracellular Matrix Constituents and Functions. Cold Spring Harbor Perspectives in Biology..

[25] Glenting J (2013). Anchorless surface associated glycolytic enzymes from Lactobacillus plantarum 299v bind to epithelial cells and extracellular matrix proteins. Microbiol Research..

[26] Kainulainen V (2012). Glutamine synthetase and glucose-6-phosphate isomerase are adhesive moonlighting proteins of Lactobacillus crispatus released by epithelial cathelicidin LL-37. Journal of Bacteriology..

[27] Ossowski VI (2010). Mucosal Adhesion Properties of the Probiotic Lactobacillus rhamnosus GG SpaCBA and SpaFED Pilin Subunits. Applied and Environmental Microbiology..

[28] Bansil R, Turner B (2006). Mucin structure, aggregation, physiological functions and biomedical applications. Current Opinion in Colloid & Interface Science..

[29] Cone R (2009). Barrier properties of mucus. Advanced Drug Delivery Reviews..

[30] Bouckaert J (2004). Receptor binding studies disclose a novel class of high-affinity inhibitors of the Escherichia coli FimH adhesin. Molecular Microbiology..

[31] Linden S, Wickström C, Lindell G, Gilshenan K, Carlstedt I (2008). Four modes of adhesion are used during Helicobacter pylori binding to human mucins in the oral and gastric niches. Helicobacter..

[32] Duary R, Batish V, Rajput Y, Grover S (2011). Assessing the adhesion of putative indigenous probiotic lactobacilli to human colonic epithelial cells. The Indian Journal of Medical Research..

[33] Zhang W (2013). Adhesive ability means inhibition activities for Lactobacillus against pathogens and S-layer protein plays an important role in adhesion. Anaerobe..

[34] Wan L, Allen K, Turner P, El-Nezami H (2014). Modulation of Mucin mRNA (MUC5AC and MUC5B) Expression and Protein Production and Secretion in Caco-2/HT29-MTX Co-cultures Following Exposure to Individual and Combined Fusarium Mycotoxins. Toxicological Sciences..

[35] Du L (2015). First two domains at the lp_1643 protein N-terminus inhibits pathogen adhesion to porcine mucus in vitro. Journal of Food Protection..

[36] Gouyer V (2001). Specific secretion of gel-forming mucins and TFF peptides in HT-29 cell of mucin-secreting phenotype. Biochimica et Biophysica Acta..

[37] Bergonzelli G (2005). GroEL of Lactobacillus johnsonii La1 (NCC 533) Is Cell Surface Associated: Potential role in interactions with the host and the gastric pathogen Helicobacter pylori. Infection and Immunity..

[38] Pijkeren VJ (2006). Comparative and Functional Analysis of Sortase-Dependent Proteins in the Predicted Secretome of Lactobacillus salivarius UCC118. Applied and Environmental Microbiology..

[39] Etzold S (2014). Structural and molecular insights into novel surface-exposed mucus adhesins from Lactobacillus reuteri human strains. Molecular Microbiology..

[40] Pacheco (2010). Viability of Lactobacillus delbrueckii Under Human Gastrointestinal Conditions Simulated In Vitro. American Journal of Agricultural and Biological Sciences.

[41] Tallon R, Arias S, Bressollier P, Urdaci MC (2007). Strain-and matrix-dependent adhesion of Lactobacillus plantarum is mediated by proteinaceous bacterial compounds. Journal of Applied Microbiology..

[42] Wampler J, Kim K, Jaradat Z, Bhunia A (2004). Heat Shock Protein 60 Acts as a Receptor for the Listeria Adhesion Protein in Caco-2 Cells. Infection and Immunity..

[43] Henderson B, Martin A (2011). Bacterial Virulence in the Moonlight: Multitasking Bacterial Moonlighting Proteins Are Virulence Determinants in Infectious Disease. Infection and Immunity..

[44] Cabanes D (2005). Gp96 is a receptor for a novel Listeria monocytogenes virulence factor, Vip, a surface protein. The EMBO Journal..

[45] Aryantini, N. P. D. *et al*. Anchorless cell surface proteins function as laminin-binding adhesins in Lactobacillus rhamnosus FSMM22. *FEMS Microbiology Letters*. **364**, 10.1093/femsle/fnx056 (2017). 10.1093/femsle/fnx056 28333282

[46] Krachler A, Orth K (2011). Functional characterization of the interaction between bacterial adhesin Multivalent Adhesion Molecule 7 (MAM7) protein and its host cell ligands. Journal of Biological Chemistry..

[47] Krachler A, Ham H, Orth K (2011). Outer membrane adhesion factor multivalent adhesion molecule 7 initiates host cell binding during infection by Gram-negative pathogens. Proceedings of the National Academy of Sciences USA.

[48] Gueimonde M, Jalonen L, He F, Hiramatsu M, Salminen S (2006). Adhesion and competitive inhibition and displacement of human enteropathogens by selected Lactobacilli. Food Research International..

[49] Torres A (2002). Characterization of Cah, a calcium-binding and heat-extractable autotransporter protein of enterohaemorrhagic Escherichia coli. Molecular Microbiology..

[50] Johnson M (2011). Pseudomonas aeruginosa PilY1 Binds Integrin in an RGD- and Calcium-Dependent Manner. PLoS ONE..

[51] Bovee-Oudenhoven I, Lettink-Wissink M, Doesburg VW, Witteman B, Meer VDR (2003). Diarrhea caused by enterotoxigenic Escherichia coli infection of humans is inhibited by dietary calcium. Gastroenterology..

[52] Wijemanne P, Moxley R (2014). Glucose significantly enhances Enterotoxigenic Escherichia coli adherence to intestinal epithelial cells through its effects on heat-labile enterotoxin production. PLoS ONE..

[53] Kolodziejek A (2013). Physiological Levels of Glucose Induce Membrane Vesicle Secretion and Affect the Lipid and Protein Composition of Yersinia pestis Cell Surfaces. Applied and Environmental Microbiology..

[54] Kinoshita H (2008). Cell surface Lactobacillus plantarum LA 318 glyceraldehyde-3-phosphate dehydrogenase (GAPDH) adheres to human colonic mucin. Journal of Applied Microbiology..

[55] George M, Abraham T (2006). Polyionic hydrocolloids for the intestinal delivery of protein drugs: Alginate and chitosan-a review. Journal of Controlled Release..

[56] Al-Qadi S, Grenha A, Carrión-Recio D, Seijo B, Remuñán-López C (2012). Microencapsulated chitosan nanoparticles for pulmonary protein delivery: In vivo evaluation of insulin-loaded formulations. Journal of Controlled Release..

[57] Baur H, Kasperek S, Pfaff E (1975). Criteria of Viability of Isolated Liver Cells. Hoppe-Seyler’s Zeitschrift für physiologische Chemie..

[58] Krieger E (2009). Improving physical realism, stereochemistry, and side-chain accuracy in homology modeling: Four approaches that performed well in CASP8. Proteins: Structure, Function, and Bioinformatics..

[59] Biasini M (2014). SWISS-MODEL: modelling protein tertiary and quaternary structure using evolutionary information. Nucleic Acids Research..

[60] Xu D, Zhang Y (2011). Improving the physical realism and structural accuracy of protein models by a two-step atomic-level energy minimization. Biophysical Journal..

[61] Luthy R, Bowie J, Eisenberg D (1992). Assessment of protein models with three-dimensional profiles. Nature..

[62] Cao E (2007). T Cell Immunoglobulin Mucin-3 Crystal Structure Reveals a Galectin-9-Independent Ligand-Binding Surface. Immunity..

[63] Howell S, Kenny AJ, Turner AJ (1992). A survey of membrane peptidases in two human colonic cell lines, Caco-2 and HT-29. Biochemical Journal..

[64] Bathla S (2015). Profiling of urinary proteins in Karan Fries cows reveals more than 1550proteins. Journal of Proteomics..

[65] Sezonov G, Joseleau-Petit D, D’Ari R (2007). Escherichia coli Physiology in Luria-Bertani Broth. Journal of Bacteriology..

