# Supplementary material for: Mechanistic insights into the host-microbe interaction and pathogen exclusion mediated by the Mucus-binding protein of Lactobacillus plantarum
Source: Sci Rep. 2018 Sep 21;8:14198. doi: 10.1038/s41598-018-32417-y (PMC6155027; doi:10.1038/s41598-018-32417-y)
Supplement: Supplementary file 1 — Supplementary information [file 41598_2018_32417_MOESM1_ESM.doc]

**Supplementary information:**

**Title:**

Mechanistic insights into the host-microbe interactions and pathogen exclusion mediated by the Mucus-binding protein of *Lactobacillus plantarum*

**Author names and affiliations**

**Kumar Siddharth Singh^1^, Sudarshan Kumar^1^, Ashok Kumar Mohanty^1^, Sunita Grover^2^, Jai Kumar Kaushik^1,3*^**

**1** Animal Biotechnology Centre, ICAR-National Dairy Research Institute, Karnal, Haryana, India;d

**2** Dairy Microbiology Division, ICAR-National Dairy Research Institute, Karnal, Haryana, India.

**3** BTIS Sub-DIC, Animal Biotechnology Centre, National Dairy Research Institute, Karnal, 132001, India.

**Corresponding author:**

**Jai Kumar Kaushik**,

**Email**: [jaikr1@gmail.com](mailto:jaikr1@gmail.com), [jai.kaushik@icar.gov.in](mailto:jai.kaushik@icar.gov.in);

**Address:** Animal Biotechnology Center, ICAR-National Dairy Research Institute, Karnal, Haryana- 132001, India.

**Table S1**: Estimation of Mubs5s6 adhesion with PGM using indirect ELISA in gastrointestinal conditions and presence of a detergent, n = 6. 7µg of Mubs5s6 protein was used in the serial numbers 3 to 7; SGF = Simulated Gastric Fluid; SIF = Simulated Intestinal Fluid, BS = Bile Salts, Tween-20 = Detergent to break hydrophobic interactions.

| **Sl. No.** | **Sample** | **% Decrease**  **O.D. (Mubs5s6 without exposure - Mubs5s6 after exposure) / Mubs5s6 without exposure x 100** | **%SEM** | **%CV** |
| --- | --- | --- | --- | --- |
| 1 | PBS (Control) | Not applicable | 0.475 | 4 to 10.5 |
| 2 | MBP (Control) | Not applicable | 0.598 | 7 to 13.2 |
| 3 | Mubs5s6_7µg (without exposure) | 0 | 2.08 | 4.22 to 7.09 |
| 4 | Mubs5s6_7µg – SGF | 35.36 | 1.07 | 4.59 |
| 5 | Mubs5s6_7µg - SIF | 11.03 | 1.24 | 3.84 |
| 6 | Mubs5s6_7µg - BS | 33.89 | 1.96 | 8.19 |
| 7 | Mubs5s6_7µg - Tween-20 | 64.63 | 1.01 | 7.88 |

**Table S2:** Effect of pH and elevated temperature on Mubs5s6 protein.

| **Sl. No.** | **pH/Buffer**  **(10mM)** | **Before heating** | **Post-heating**  **(up to 95°C)** | **Observation** |
| --- | --- | --- | --- | --- |
| 1 | 2.0/Glycine | Aggregation observed | X | Unstable even at room temp. |
| 2 | 3.5/Acetate | Aggregation observed | X | Unstable even at room temp. |
| 3 | 5.0/Acetate | Aggregation observed | X | Unstable even at room temp. |
| 4 | 7.4/Tris-HCl | Clear/transparent | Aggregation observed after 50°C | Irreversible denaturation |
| 5 | 8.0/Tris-HCl | Clear/transparent | Aggregation observed after 50°C | Irreversible denaturation |
| 6 | 10.0/Glycine | Clear/transparent | Aggregation observed after 50°C | Irreversible denaturation |

**Table S3:** Decrease in adhesion of enterotoxigenic *E. coli* (ETEC) to HT-29 cells and Caco-2 cells in the presence of Mubs5s6 protein or *L. plantarum*, n = 6. Here, Mubs5s6 protein refers to the total Mubs5s6 protein released from encapsulated chitosan-TPP microspheres after incubation for 20 hours in simulated intestinal buffer. The probiotic *L. plantarum* Lp9 and pathogen ETEC cells were used at 10^6^ CFU/ml.

| **Sl. No** | **Cell line: Mubs5s6 or *L. plantarum* Lp9: ETEC** | **%Decrease ± SEM** |
| --- | --- | --- |
| **1** | HT-29: No protein or Lp9: ETEC (control) | 0 |
| **2** | HT-29: Mubs5s6 (25 µg/ml): ETEC | 32.8 ± 0.8 |
| **3** | HT-29: Mubs5s6 (125 µg/ml): ETEC | 52.98 ± 0.7 |
| **4** | HT-29: Mubs5s6 (400 µg/ml): ETEC | 80.4 ± 0.9 |
| **5** | HT-29: Lp9: ETEC | 49.15 ± 0.9 |
| **6** | Caco-2: No protein or Lp9: ETEC (control) | 0 |
| **7** | Caco-2: Mubs5s6 (25 µg/ml): ETEC | 23.74 ± 0.8 |
| **8** | Caco-2: Mubs5s6 (125 µg/ml): ETEC | 38.79 ± 0.7 |
| **9** | Caco-2: Mubs5s6 (400 µg/ml): ETEC | 67.17 ± 0.6 |
| **10** | Caco-2: Lp9: ETEC | 35.9 ± 0.9 |

**
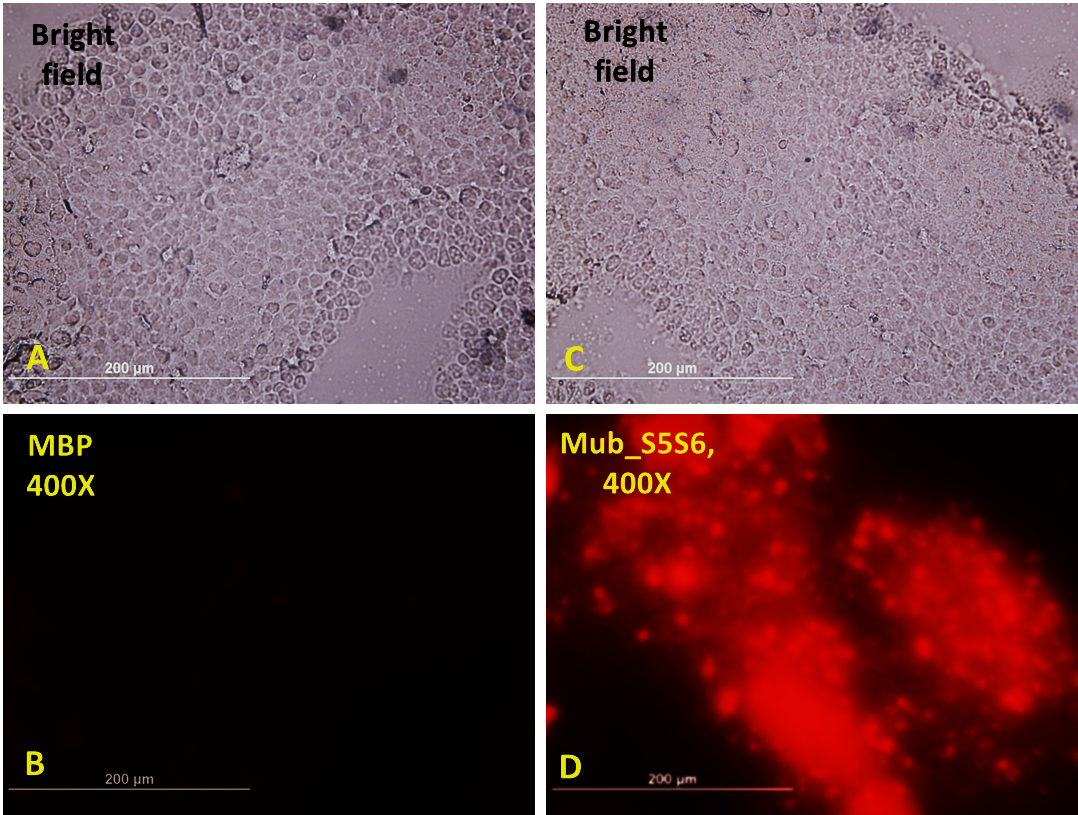
**

**Figure S1**: Adhesion of MBP-Mubs5s6 with HT-29 intestinal epithelial cell line **A)** Bright field, 400X of Control **B)** Immunostained, 400X of Control **C)** Bright field, 400X of Mubs5s6 **D)** Immunostained, 400X of MBP-Mubs5s6.

**
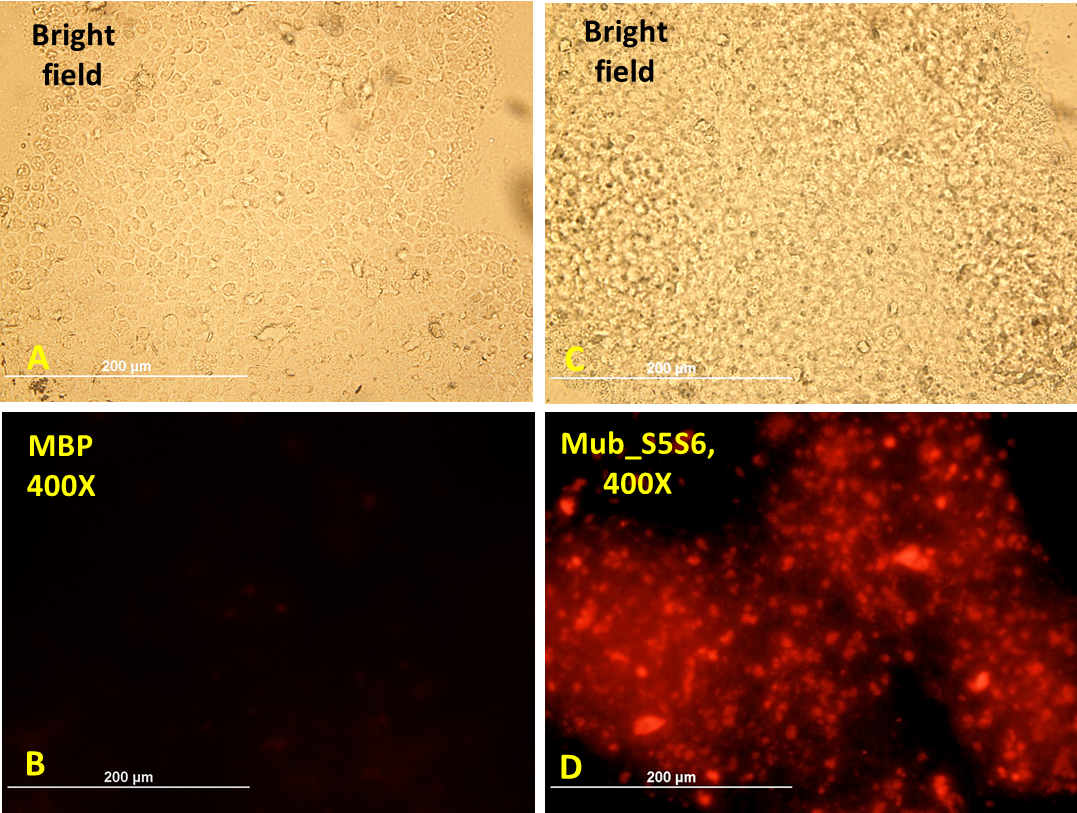
**

**Figure S2**: Adhesion of MBP-Mubs5s6 with Caco-2 intestinal epithelial cell line **A)** Bright field, 400X of Control **B)** Immunostained, 400X of Control **C)** Bright field, 400X of Mubs5s6 **D)** Immunostained, 400X of MBP-Mubs5s6.


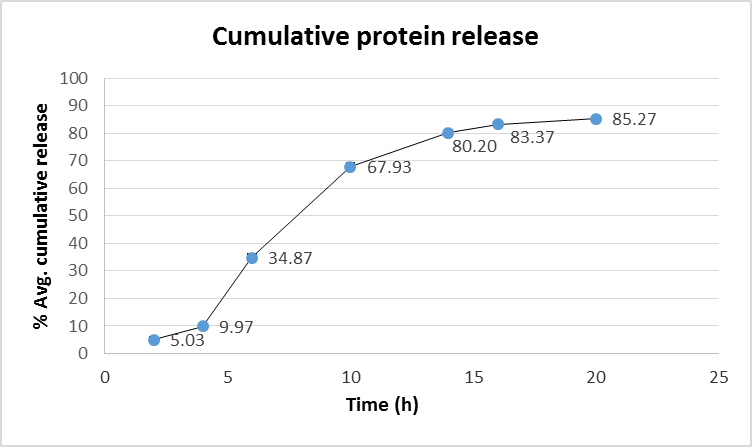


**Figure S3**: Percentage average controlled release of Mubs5s6 protein from Chitosan-TPP microspheres over a period of 20 hours (2 hours in pH 1.2 buffer and 18 hours in pH 6.8 buffer).


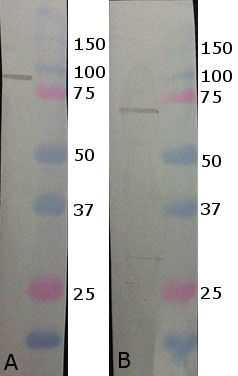


**Figure S4**: Western blot to confirm the presence of A) Hsp90 and B) Laminin alpha 2, in the pull-down eluates of >60kDa fractions. Hsp90 shows single band while Laminin shows 2 bands with a prominent band more than 60kDa and smaller faint band of about 30kDa size. These might be degradation products of Laminin alpha 2 which has average size more than 200kDa.

**Protocol used for binding of Mubs5s6 to Calcium and Glucose**

Atomic absorption spectrophotometer reference stock is generally prepared using CaCO_3_ in slightly acidic solution by using 0.5 ml of 6 M HCl per liter. Acidic CaCO_3_ solution could not be used in our calcium binding experiments to avoid denaturation of MBP tag removed Mubs5s6 protein. Therefore, we used calcium chloride as it could be completely solubilized at high concentrations in deionized water. Mubs5s6 protein was mixed with 1000 ppm of aqueous calcium chloride and left at 37ºC for 2 hours. The mixture was extensively dialyzed against MilliQ water with three changes each at every six hours. The Mubs5s6-Ca complex was mixed with 50 ppm lanthanum chloride (to minimize interference of phosphorus), filtered through 0.2 µm filter and this sample was measured for the content of bound calcium using a ZEEnit 700 P Atomic Absorption Spectrophotometer (Analytik Jena, Germany) in flame mode. BSA was used as a negative control since it is a eukaryotic protein with no known and predicted calcium binding activity. Binding of dextrose (1 mg/ml) with Mubs5s6 protein was studied in the presence of 10 mM Tris-HCl buffer (pH 7.4). The bound amount of glucose was estimated using Glucose Assay kit (CBA086, Merck, Germany). All the measurements were carried out at least three times in duplicates.
